# Human Stem Cell-Derived Neurons: A System to Study Human Tau Function and Dysfunction

**DOI:** 10.1371/journal.pone.0013947

**Published:** 2010-11-11

**Authors:** Mariangela Iovino, Rickie Patani, Colin Watts, Siddharthan Chandran, Maria Grazia Spillantini

**Affiliations:** 1 Department of Clinical Neurosciences, Cambridge Centre for Brain Repair, University of Cambridge, Cambridge, United Kingdom; 2 Anne McLaren Laboratory for Regenerative Medicine, University of Cambridge, Cambridge, United Kingdom; 3 Centre for Clinical Brain Sciences, MRC Centre for Regenerative Medicine, University of Edinburgh, Edinburgh, United Kingdom; New York State Institute for Basic Research, United States of America

## Abstract

**Background:**

Intracellular filamentous deposits containing microtubule-associated protein tau constitute a defining characteristic of many neurodegenerative disorders. Current experimental models to study tau pathology *in vitro* do not usually recapitulate the tau expression pattern characteristic of adult human brain. In this study, we have investigated whether human embryonic stem cell-derived neurons could be a good model to study human tau distribution, function and dysfunction.

**Methodology/Principal Findings:**

Using RT-PCR, immunohistochemistry, western blotting and cell transfections we have investigated whether all 6 adult human brain tau isoforms are expressed in neurons derived from human embryonic and fetal stem cells and whether 4 repeat tau over-expression alone, or with the F3 tau repeat fragment, (amino acid 258–380 of the 2N4R tau isoform with the ΔK280 mutation) affects tau distribution. We found that the shortest 3 repeat tau isoform, similarly to human brain, is the first to be expressed during neuronal differentiation while the other 5 tau isoforms are expressed later. Over expression of tau with 4 repeats affects tau cellular distribution and the short tau F3 fragment appears to increase tau phosphorylation but this effect does not appear to be toxic for the cell.

**Conclusions:**

Our results indicate that human embryonic stem cell-derived neurons express all 6 tau isoforms and are a good model in which to study tau physiology and pathology.

## Introduction

Tau is a microtubule-associated protein involved in microtubule assembly, stabilization and axonal transport [1], [2]. The human tau gene is located in chromosome 17, it consists of 16 exons that are alternatively spliced to generate six different tau isoforms. These isoforms differ for the presence of 3 or 4 tandem repeats (3R,4R) in the carboxy-terminal region and 0 (0N), 29 (1N) or 58 (2N) amino-acid inserts in the amino-terminal part of the protein [3]. In fetal human brain only the shortest isoform with 0N3R is expressed [4] while in normal adult human brain all six tau isoforms are present and the ratio between 3R and 4R isoforms is approximately 1∶1. This ratio is important since at equal total amount of tau, an imbalance in the expression of 3R and 4R tau causes frontotemporal dementia and parkinsonism linked to chromosome 17 (FTDP-17T) [5]. The functional differences between the 3R and 4R tau isoforms and the reasons why one cannot replace the other are not known. Current experimental models for studying tau and its pathology do not recapitulate the tau pattern of expression observed in adult human brain. Human embryonic stem cells (hESCs) could be a reproducible model to study the expression and localization of 3R and 4R tau as well as to investigate the effects of 4R tau over-expression and tau aggregation. Here we report that hESC-derived neurons express all 6 tau isoforms and represent a good system to study human tau function and dysfunction.

## Materials and Methods

### Cell Culture

All studies and tissue procurement were approved by the Cambridge Local Regional Ethics committee. For neuronal primary cultures written consent was obtained from each patient to use tissue removed as part of a surgical procedure in accordance with section 16 [2] (e) (ii) of the United Kingdom Human Tissue Act. hESC lines H9 and HuES9 were obtained from the WiCell Research Institute (Madison, WI) and the hESCs facility of Harvard University (Cambridge, MA) respectively. Differentiation of hESCs into neurons started between passages 30 and 70 of the original lines. hESC cultures were differentiated into neurons using a modified published protocol [6], [7]. Briefly, hESCs were propagated in defined medium supplemented with 8 ng/ml FGF2, 10 ng/ml Activin and 10 ng/ml insulin. To generate neurospheres containing neuronal stem cells, hESCs were washed in phosphate buffered saline (PBS), then dissociated from the underlying mouse embryonic fibroblast feeder layer by gentle pipetting. Colonies were mechanically triturated before being plated at a low density in 10 cm culture dishes on an orbital shaker. Cells were then maintained in defined medium in the presence of 20 ng/ml of FGF2 from day 8 [7]. To differentiate neuronal stem cells into neurons, neurospheres were plated on glass coverslips coated with 10 µg/ml poly-L-lysine and 10 µg/ml laminin (Sigma-Aldrich, Dorset UK) and kept in differentiating medium (DMEM, 2% B27, 1% PSF, BDNF and GDNF) for several weeks.

Fetal stem cells (hFSCs) neuronal cultures were established from 12–14 weeks old human post-mortem fetal tissue. Briefly, tissue was dissociated into a single-cell suspension by incubation with accutase (PAA laboratories, Linz, Austria) at 37°C for 15 min and mechanically dissociated by using a fire-polished Pasteur pipette. Cells were grown for several days in DMEM, containing F12, 2% B27, 1% PSF, EGF and FGF, in order to form neurospheres that were then dissociated with accutase and single-cells were plated as for hESCs and at a density of 5×10^4^ cells per well. Cells were grown for several weeks in DMEM containing 2% B27, 1% PSF to differentiate them into neurons.

Adult neurons were derived from normal temporal cortical samples removed during surgery for medial temporal lobe low grade glioma. Tissue was dissected into small pieces and plated in DMEM containing 2% B27, 1% PSF on glass-coverslips previously covered with extracellular matrix peptides (ECM; Sigma-Aldrich). After 48 hrs at 37°C in 5% CO^2^ tissue was removed from coverslips and neurons growing from the explants were fixed in 4% PFA.

### RNA extraction and RT-PCR

Total RNA was extracted from hESC- or hFSC-derived neurons and adult human brain tissue with the RNeasy mini kit (Qiagen, Crawley, UK) according to the manufacture's protocol. Three repeat and 4R tau mRNA expression was detected by RT-PCR using One step RT-PCR kit (Qiagen,Crawley, UK) and primers: forward 5′-AAGTCGCCGTCTTCCGCCAAG-3′; reverse 5′-GTCCAGGGACCCAATCTTCGA-3′. Glyceraldehyde-3-phosphate dehydrogenase (GAPDH, primers: forward 5′-CCATGGCACCGTCAAGGCTGA-3′; reverse 5′- GCCAGTAGAGGCAGGGATGAT-3′) was used as a control gene. PCR conditions were as follows: 15 min at 95°C, and then 30 cycles of 30 sec at 94°C, 30 sec at 60°C, 1 min at 74°C with a final 10 min extension at 72°C. RT-PCR products were detected on 1.5% agarose gel: 4R and 3R tau RT-PCR products were 381 and 288 bp respectively.

### Immunofluorescence

Neurons derived from hESCs, hFSCs and adult human brain biopsy were fixed in 4% paraformaldehyde for 20 min at room temperature and treated with 5% goat serum in PBS/0.01% Tween-20 for 1 hr. The following monoclonal and polyclonal primary antibodies were used: anti-3R tau (clone RD3, Upstate, Billerica, MA, USA; 1∶500), anti-4R tau (clone RD4, Upstate; 1∶500), phosphorylation-dependent anti-tau antibodies AT8 (Innogenetics, Gent, Belgium, 1∶1000), pSer262 (Invitrogen,1∶1000), AT100 (Innogenetics, Gent, Belgium, 1∶1000), anti-βIIItubulin (Millipore,Billerica, MA, USA 1∶1000), anti-MAP2 (clone HM2; Sigma-Aldrich; 1∶500); anti-neurofilament antibody (Chemicon, Temecula, CA, USA, 1∶1000), polyclonal antiserum anti-βIIItubulin (Covance, Princenton NJ, USA, 1∶1000), anti-exon10 tau (E10, kind gift from N. Sergeant, INSERM, Lille, France; 1∶400) and polyclonal human specific total tau antibody (Dako,Glostrup,Denmark, 1∶1000). Cells were incubated overnight at 4°C with the various primary antibodies then washed in PBS and incubated for 1 hr with fluorescent-conjugated secondary antibodies (Alexa, Invitrogen Molecular Probes, Eugene OR, USA) at room temperature. For 3R and 4R tau immunofluorescence, cells were incubated with biotinylated secondary antibodies (1∶250) for 2 hrs at room temperature and 1 hr with fluorescent streptavidin (Alexa Invitrogen, 1∶500).

Double-labeling immunofluorescence was performed by sequential incubation with the appropriate primary antibodies. Cell nuclei were visualized with DAPI (Sigma Aldrich, 1∶10,000).

### Immunoblotting

hESC- and hFSC-derived neurones were scraped from the plate in PBS/EDTA. Soluble tau was extracted from cells with 2.5% perchloric acid (Sigma-Aldrich) as previously described [4]. Dephosphorylation of tau was performed with 0.3 units/µl of Alkaline Phosphatase from *Escherichia coli* (Sigma-Aldrich) in 50 mM Tris-HCl and 5mM MgCl2 for 3–4 hrs at 65°C. Proteins were separated on 10% SDS-PAGE and transferred onto Immobilon-P membranes (Millipore, Bedford, MA, USA). Membranes were incubated for 1 hr with 5% w/v Marvel milk (Premier International Foods, Spalding, UK) in TBS/0.01% Tween-20 then probed with polyclonal anti-human tau antiserum (Dako, Glostrup, Denmark 1∶1000) followed by peroxidase-conjugated secondary antibody (Dako, 1∶10,000). Blots were developed using Western Lightining Chemioluminescence Reagent Plus (Perkin Elmer, Waltha, MA, USA).

### DNA Cloning and Transfection

Human tau43 (0N4R isoform) was cut out from the vector pSG5Tau43 (kind gift from M. Goedert, LMB-MRC Cambridge, UK) and cloned into the pIRES2dsred2 vector (Invitrogen, Eugene OR, USA). The F3 fragment, corresponding to amino acid sequence 258–380 of the longest human tau isoform and containing the ΔK280 mutation [8], was amplified from the plasmid pRK172tau43ΔK280 (kind gift of M. Goedert, MRC-LMB Cambridge, UK) by PCR (primers: forward 5′-AGACGGATCCGCCACCAT GTCCAAGATCGGCTCCACT-3′; reverse 5′-AGACGCGGCCGCTCAGATATTGTCC AGGG ACCCAAT-3′) and sub-cloned into pcDNA3 vector. hESCs-derived neurons were transfected with empty vector or 0N4R tau (0.8 µg DNA) and/or F3 plasmid (0.8 µg DNA) using Lipofectamine2000 according to manufacture's protocol (Lipofectamine 2000, Invitrogen).

### Propidium iodide staining

Propidium iodide (PI) staining was performed 72 hrs after cell transfection. Cells were treated with 1 µl of 2 mg/ml PI (Sigma-Aldrich), incubated at 37°C for 10 min, washed twice with PBS and then fixed with 4% PFA for 10 min. Dead cells were visualized under the microscope using a 568 filter. A total of 1000 cells (identified by DAPI staining) per coverslip were counted. Two coverslips were counted for each condition in 2 separate experiments.

## Results

### Adult human brain tau isoforms are expressed in hESC-derived neurons

**Figure 1:**
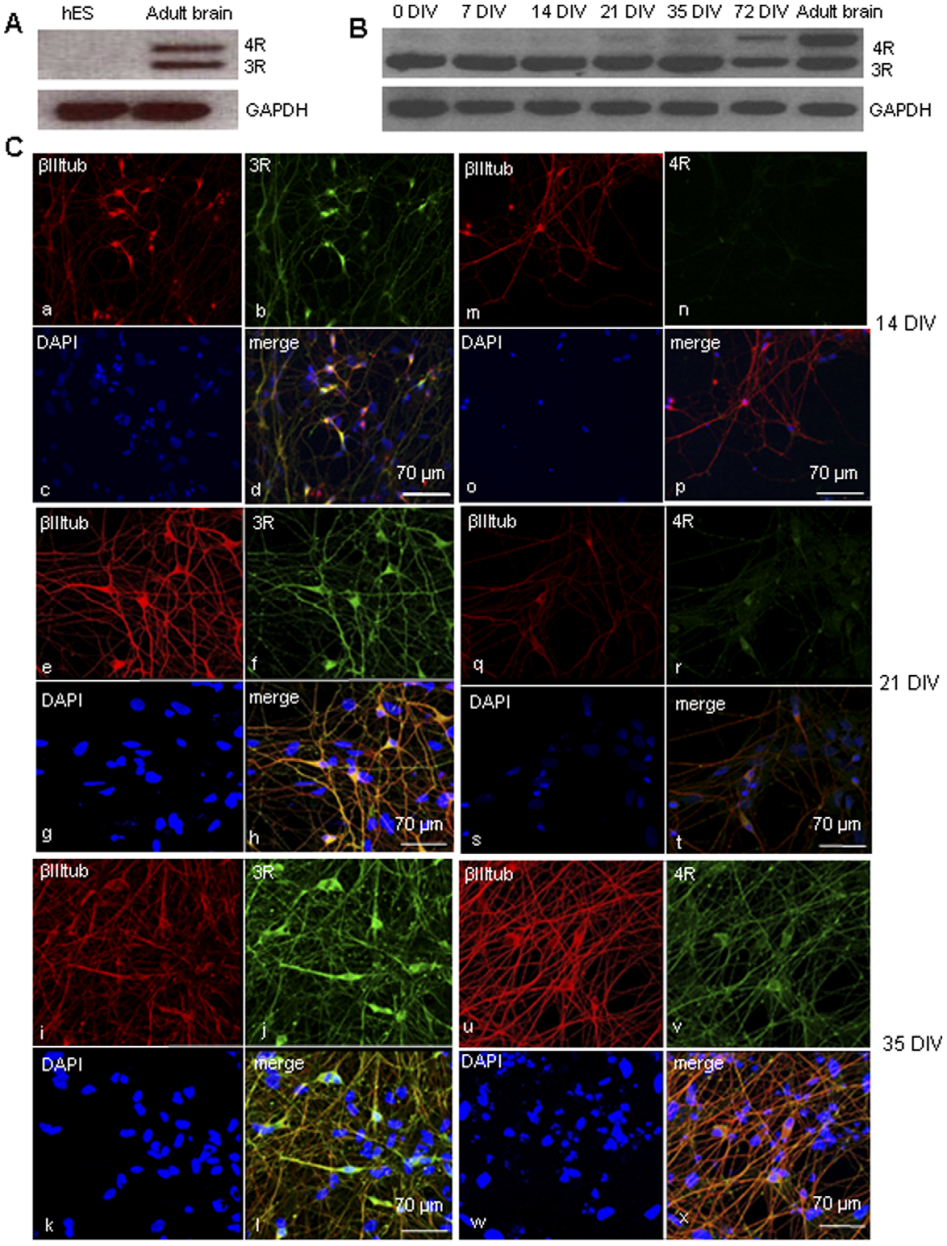
Tau expression in hESC-derived neurons. **(A)** RT-PCR for 3R and 4R tau isoforms in hESCs before differentiation (hES) and in adult human brain. hESCs do not express tau protein while in the adult human brain the ratio between 3R and 4R tau mRNA is about 1∶1. **(B)** RT-PCR for 3R and 4R tau in hESCs at 7 DIV, 14 DIV, 21 DIV, 35 DIV, 72 DIV and adult brain tissue shows that 4R tau increases during neuronal differentiation. **(C)** (a–l): immunocytochemistry for 3R tau (green), β-IIItubulin (red) and DAPI (blue) after 14 DIV (a–d), 21 DIV (e–h) and 35 DIV (i–l). 3R tau co-localizes with β-IIItubulin and is strongly expressed in neuronal cell bodies and axons. (m–x): Immunocytochemistry for 4R tau (green), β-IIItubulin (red) and DAPI (blue) at 14 DIV (m–p), 21 DIV (q–t) and 35 DIV (u–x). Four repeat tau is not expressed at 14 DIV, but it starts to appear in cell bodies at 21 DIV. The expression of 4R tau becomes stronger at 35 DIV when is present also in axons. RD3 and RD4 antibodies were used to detect 3R and 4R tau.

**Figure 2:**
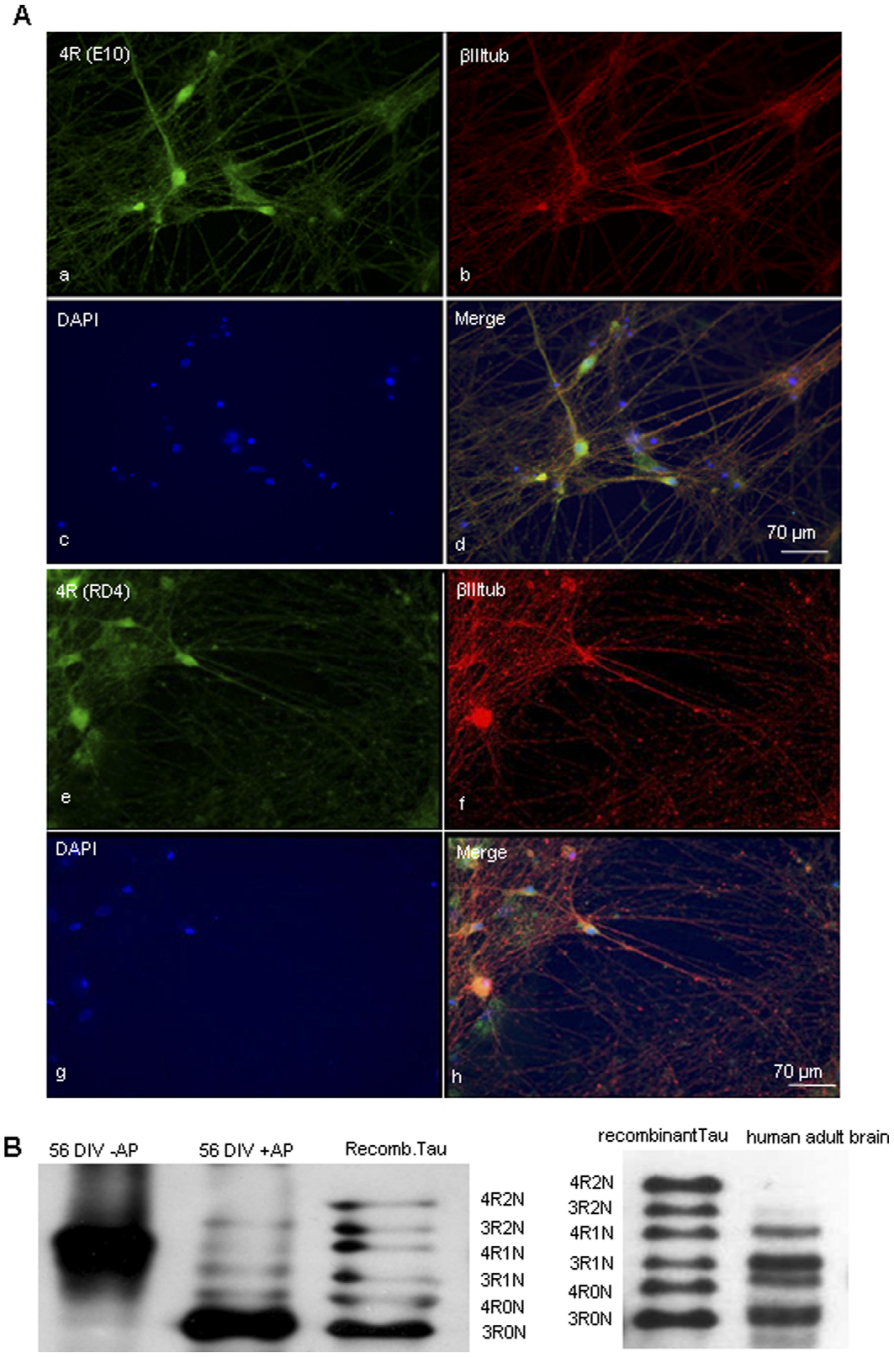
Tau expression in hESC-derived neurons after 72 days in culture. **(A)** Immunostaining with anti-4R tau antibodies E10 (a–d) and RD4 (e–h) shows that 4R tau isoforms are present in neuronal cell bodies and axons. Both antibodies are 4R tau isoform-specific. **(B)** Tau isoform expression in hESC-derived neurons at 56 DIV. The immunoblot (human tau antibody, Dako) shows the tau isoforms before and after alkaline phosphatase (AP) treatment compared to human recombinant tau. Bands corresponding to the 6 tau isoforms can be seen and the shortest 3R tau isoform is the most abundant. De-phosphorylated tau extracted from adult human brain is also shown. The data are representative of at least three independent experiments. Similar results were obtained in both H9 and HuES cell lines.

hESCs were differentiated first into NSCs and then into human neurons as described [6], [7]. RT-PCR of 3R and 4R mRNA detected at different stages of differentiation showed no tau mRNA expression in undifferentiated hESCs (Fig 1A) with tau mRNA appearing at 7 DIV and after at 14, 21, 35 and 72 DIV (Fig 1B). Similarly to human fetal brain, 3R tau was the first to be expressed while the expression of 4R tau started later and increased with neuronal maturation [4] (Fig 1B). Immunofluorescence with the isoform-specific RD3 and RD4 antibodies confirmed that 3R tau protein was detectable from 14 DIV while 4R tau was present from 21 DIV. Three repeat tau was expressed in both cell bodies and axons, and its staining increased at 21 DIV and 35 DIV (Fig 1C a–l). In contrast, at 21 DIV 4R tau was only present in cell bodies, and consistently appeared in axons only at 35 DIV (Fig 1C m–x). Immunofluorescence at 72 DIV with E10 and RD4 antibodies showed that 4R tau is distributed in cell bodies and axons of neurons stained by βIII tubulin (Fig 2A). The axonal localization of 3R and 4R tau was confirmed by MAP2 and neurofilament stainings (data not shown). Immunoblot analysis of 56 DIV hESC-derived neurons showed expression of all six tau isoforms similarly to adult human brain but with the 0N3R isoform more abundant (Fig 2B).

### Tau isoform expression occurs earlier in human fetal-derived neurons

**Figure 3:**
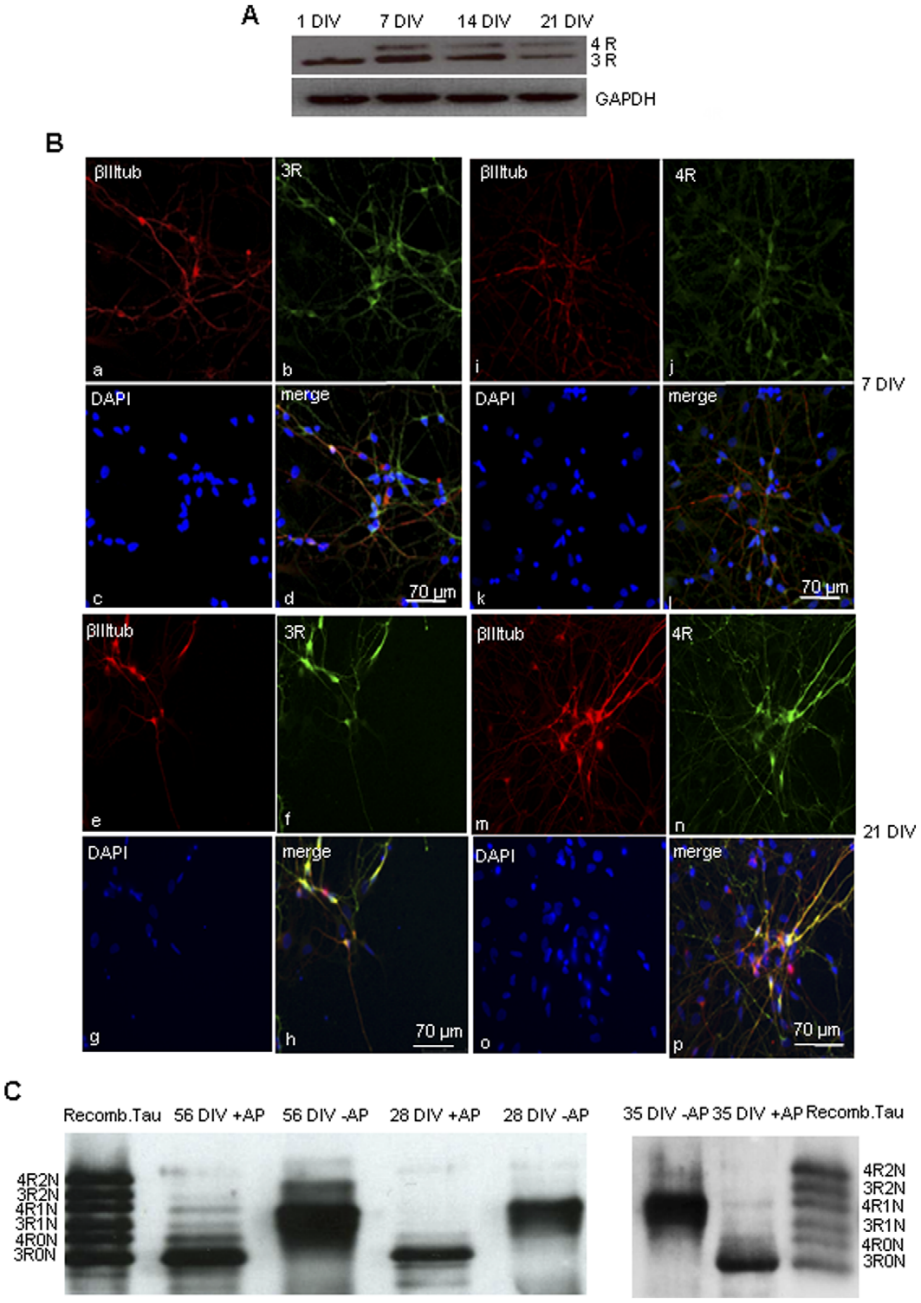
Tau expression in hFSC-derived neurons. **(A)** RT-PCR for 3R and 4R tau in hFSCs after 1 DIV, 7 DIV, 14 DIV and 21 DIV. Four repeat tau mRNA appears after 7 DIV and increases during neuronal differentiation. At 21DIV the level of expression of 3R tau is reduced. **(B)** (a–h): immunocytochemistry for 3R tau (green), β-IIItubulin (red) and DAPI (blue) in hFSC-derived neurons at 7 DIV (a–d) and 21 DIV (e–h). At 7 DIV 3R tau is strongly expressed in both cell bodies and axons as seen also at 21 DIV. (i–p): immunocytochemistry for 4R tau (green), β-IIItubulin (red) and DAPI (blue) in neurons at 7 DIV and 21 DIV. At 7 DIV 4R tau is visibile in cell bodies while at 21 DIV is present in both cell bodies and axons. **(C)** Immunoblot of tau isoforms extracted from hFSC-derived neurones. At 28 DIV only the two shortest 3R and 4R isoforms are visible while at 35 DIV and 56 DIV all six tau isoforms are present. Anti-human tau antibody (Dako, 1∶1000) is used for immunoblotting.

**Figure 4:**
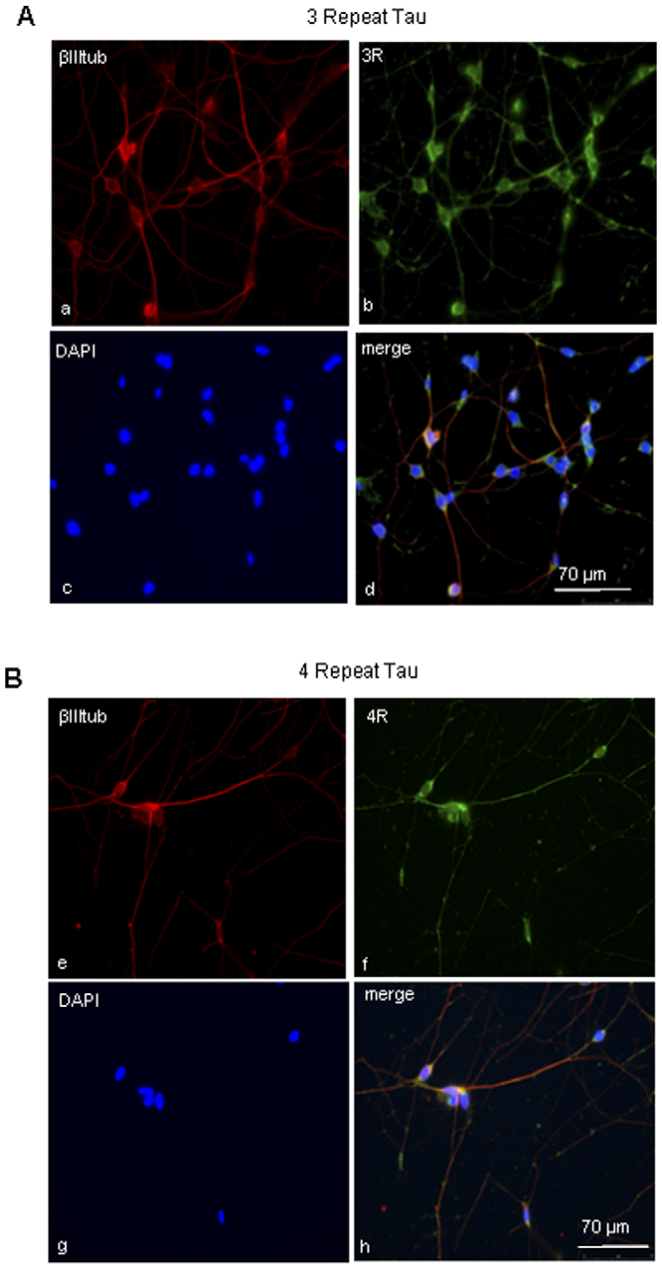
Tau expression in adult brain biopsy: **(A)** (a–d): immunocytochemistry for 3R tau (green), β-IIItubulin (red) and DAPI (blue) and **(B)** (e–h) for 4R tau (green), β-IIItubulin (red) and DAPI (blue) in primary culture of biopsy derived human adult neurons. Both isoforms co-localize with β-IIItubulin and are similarly expressed in neuronal cell bodies and axons.

**Figure 5:**
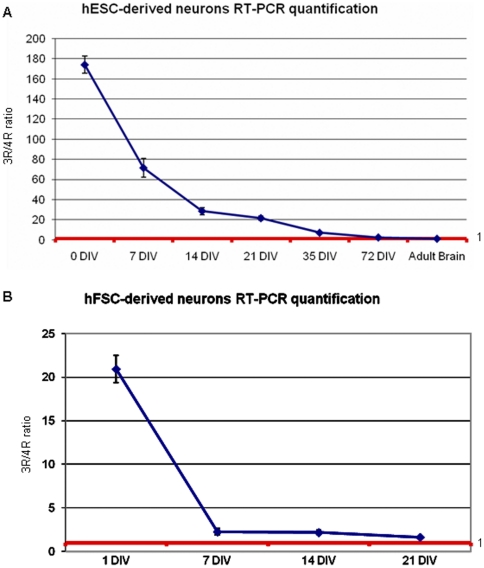
RT-PCR quantification of tau isoform expression. **(A)** hESC-derived neurons: quantification (Image J software) of 3R and 4R tau RT-PCR bands shows that the ratio between 3R and 4R tau isoforms becomes similar to that in adult human brain [1] during neuronal differentiation. **(B)** hFSC-derived neurons: quantification (Image J software) of 3R and 4R tau RT-PCR bands shows that the time for differentiation of neurons derived from hFSCs is shorter than that for hESCs, at 21 DIV the ratio between 3R and 4R tau isoforms is already similar to that in adult human brain [1].

EGF/FGF-expanded neural precursors derived from first trimester fetal human nervous tissue were cultured in order to determine tau isoform expression in cells that are more advanced compared to hESCs in their neuronal commitment pathways prior to differentiation. RT-PCR showed 3R tau mRNA already at 1 DIV while 4R tau mRNA was present only at 7 DIV (Fig 3A). Three and 4R tau protein expression was detected by immunofluorescence from 7 DIV, in contrast to hESC-derived neurons, where expression of 4R tau started at 21 DIV. Confocal microscopy examination at 21 DIV showed 4R tau already distributed along the axons (Fig 3Bm,p). Immunoblot analysis of hFSC-derived neurons showed that only the shortest 3R and 4R tau isoforms lacking amino-terminal inserts were expressed at 28 DIV, while at 35 DIV and 56 DIV, all 6 tau isoforms were present similar to human adult brain (Fig 3C) but with 0N3R more abundant. The distribution and expression of 3R and 4R tau isoforms in hESC- and hFSC-derived neurons was similar to that in neurons from human adult brain biopsy primary cultures (Fig 4). Furthermore, the 3R and 4R tau 1∶1 ratio, like that present in adult human brain, was reached in both neurons derived from hESCs and hFSCs upon differentiation (Fig 5).

### Four repeat tau over-expression causes a premature distribution of tau into axons

**Figure 6:**
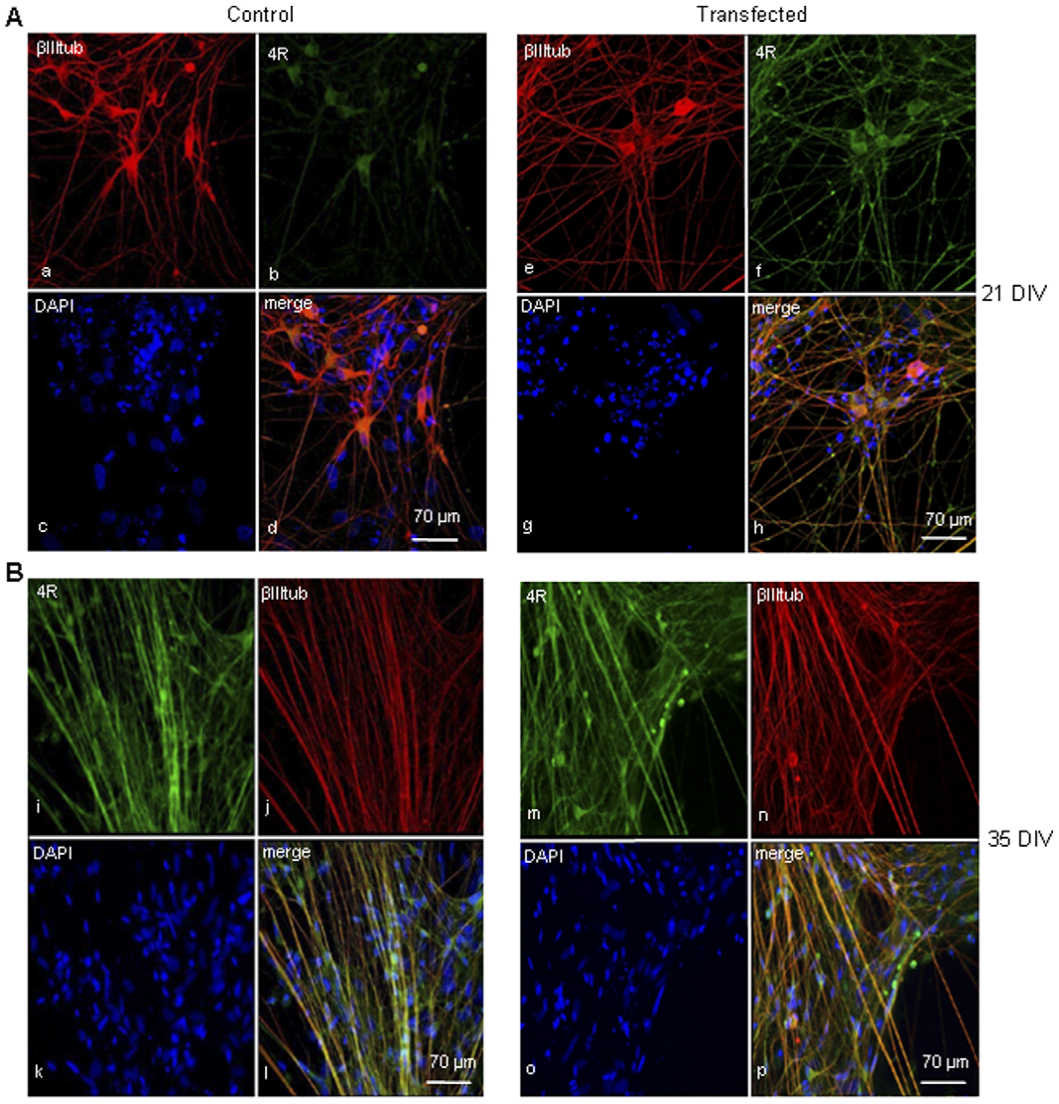
Four repeat tau over-expression (A–B). hESC-derived neurons transfected with the shortest 4R tau (0N4R) at 21 DIV **(A)** and 35 DIV **(B)**. **(A)**: Immunocytochemistry for 4R tau (green), β-IIItubulin (red) and DAPI (blue) in control cells (a–d) and in transfected cells (e–h). Immunocytochemistry shows that in transfected cells 4R tau is present also in the axons of neurons and not only in the cell bodies as in control cells. **(B)**: There is no difference in the distribution of 4R tau in control cells (i–l) and in 4R tau transfected cells (m–p) when transfection is performed at 35 DIV although, in axons of transfected cells the intensity of fluorescence is increased. Immunocytochemistry with anti-4R tau antibody (E10) is performed at 72 hrs after transfection.

To further characterize the time course of 4R tau localization when overexpressed, hESC-derived neurons were transfected with the shortest 4R tau (htau-43,0N4R). In control cells at 21 DIV, endogenous 4R tau was present only in cell bodies (Fig 6A a–d) while in cells transfected with 0N4R, tau was already present along the axons (Fig 6A e–h) and differently from untransfected cells staining showed a stippled labeling. A clear re-distribution of 4R tau was not observed in hESC-derived neurons transfected with 4R tau at 35 DIV when endogenous 4R tau is already present in cell bodies and axons (Fig 6B m–p). However, axons of transfected neurons (Fig 6B m–p) were more fluorescent compared to control neurons (Fig 6B i–l).

### The F3 fragment promotes phosphorylation of endogenous and transfected 4R tau

**Figure 7:**
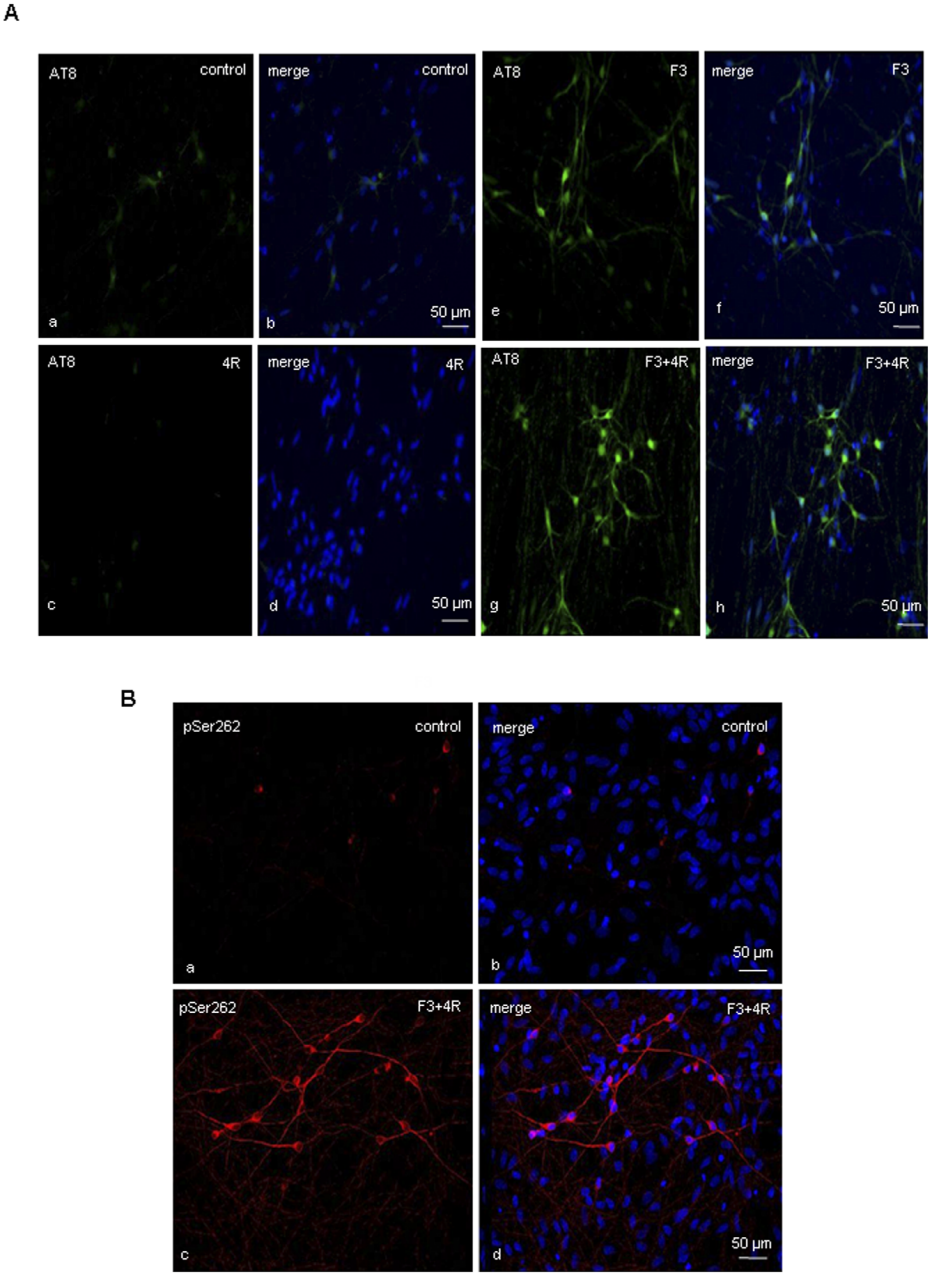
F3 fragment induces tau hyperphosphorylation and redistribution. **(A)** Immunostaining with AT8 antibody in hESC-derived neurons transfected at 35 DIV with the shorter 4R tau isoform (c–d) and empty vector (a–b). The over-expression of 4R tau isoform does not increase tau hyperphosphorylation. **(e–h)** Immunostaining with AT8 antibody in hESC-derived neurons co-transfected at 35 DIV with F3 fragment and 0N4R tau (g–h) and cells transfected only with F3 fragment (e–f). An increase in AT8 staining is present in cells transfected with only F3 (e,f) compared to controls (a,b). Neurons co-transfected with F3 and 0N4R show tau hyperphosphorylation and more dotted appearance in processes (g,h). **(B)** Immunostaining with pSer262 anti-tau antibody in hESC-derived neurons co-transfected with F3 fragment and 0N4R tau at 35 DIV: neurons co-transfected with F3 fragment and 0N4R (c–d) show an increase in pSer262 staining compared to control cells (a–b).

The F3 fragment, previously shown to promote tau aggregation and nucleation [8], was co-transfected with 0N4R tau into hESC-derived neurons at 35 DIV. Control cells were transfected with vectors containing the F3 fragment or 4R tau, or empty vectors. Cells were fixed 72 hrs after transfection and stained with anti-phospho-tau antibodies AT8, pSer262 and AT100. Cells transfected with an empty vector or 4R tau alone showed weak staining in cell bodies (Fig 7A a–d). F3 fragment-transfected cells showed increased staining in both cell bodies and axons (Fig 7A e,f), indicating that the presence of the F3 fragment is sufficient to increase endogenous tau hyperphosphorylation, staining with a total tau antibody showed similar level of tau in cells transfected with an empty vector or the F3 fragment (data not shown) excluding the possibility that the increase of phosphorylation is related to an increase of tau level. AT8 and pSer262 stainings were greatly increased in cells co-transfected with the F3 fragment and 4R tau (Fig 7A g–h; Fig 7B). No staining was observed using thioflavin S or FSB or AT100 suggesting the absence of tau filaments. No cell damage was observed with propidium iodide staining.

## Discussion

The finding that Tau gene mutations cause FTDP-17T demonstrates that alterations in tau lead to neuronal cell death and disease [9], [10], [11]. In particular, FTDP-17T mutations that alter splicing of exon 10 usually result in over-expression of 4R over 3R tau isoforms without changing the total tau content, indicating that 4R tau does not simply replace the function of 3R tau. The functional difference between these isoforms is not clear. To understand the pathway linking 4R tau over-expression to neurodegeneration, there is a need to identify an appropriate model where the pattern of tau isoform expression is comparable to that of adult human brain. The absence of a consistent amount of 3R tau in the adult rodent brain makes it unsuitable to address questions related to the imbalance between 3R and 4R tau. In human neuroblastoma cell lines such as SH SY5Y, it is difficult to obtain an adult tau isoform pattern; even after several weeks of differentiation, the shortest 3R and 4R isoforms are the predominant species. Furthermore, transgenic mice expressing the entire human tau gene also do not show the tau isoform expression pattern found in adult human brain [12], [13]. Against this background, hESC-derived neurons could be an alternative *in vitro* model to study tau physiology and pathology. We have studied the temporal distribution and expression of 3R and 4R tau isoforms at different stages of hESCs neuronal differentiation to determine whether neurons with the six tau isoforms and an appropriate tau pattern can be obtained upon maturation. We found that undifferentiated hESCs do not express tau and, as expected, the shortest 3R tau isoform, also known as fetal tau, is the first tau to be expressed at the beginning of neuronal differentiation. The transition from 3R tau to the more complex adult pattern with approximately equal amounts of 3R and 4R tau isoforms is developmentally regulated with 4R tau expression increasing according to neuronal maturation. Immunofluorescence of hESC-derived neurons at 14 DIV showed only 3R tau in neuronal cell bodies and axons and its level remained strong also at 21 and 35 DIV. In contrast, 4R tau appeared after 21 DIV, it was initially restricted to cell bodies with axonal expression becoming evident only at 35 DIV. At 56 DIV, all six tau isoforms were expressed in a pattern similar to neurons derived from adult human brain biopsy or extracted from human brain, although the shortest 3R tau appeared somehow more abundant. Keeping this in mind we can say that neurons derived from hESCs having all six tau isoforms could be a suitable system to study an imbalance between 3R and 4R tau.

hFSCs derived from 9–12-week old fetal brains represent a more advanced stage of neuronal commitment and accordingly expressed earlier tau mRNA as well as 3R and 4R tau protein isoforms with 3R tau present at 1 DIV and both 3R and 4R tau at 7 DIV. However, like in hESC-derived neurons, 3R tau was initially in both neuronal cell bodies and axons, while 4R tau was weakly expressed only in cell bodies. Only later 3R and 4R tau were similarly expressed in both cell bodies and axonal processes and their levels of expression were similar, as indicated by RT-PCR. Moreover, immunoblot analysis showed that at 35 DIV all six adult human brain tau isoforms were present. This pattern was unchanged at 56 DIV, indicating that the process of differentiation was complete. Again, when all six tau isoforms were present, the 0N3R tau isoform was more abundant compared to human brain, consistent with the tau isoform expression pattern in hESC-derived neurons. The increase of 0N3R tau in these cellular systems could be due to the fact that not all cells were at the same stage of differentiation and those at earlier stages expressed more 0N3R tau when compared with other isoforms, thus contributing to the difference with human adult brain where neurons are fully differentiated.

While 3R tau was usually seen in both cell bodies and axons, 4R tau was initially seen in cell bodies and only after some time in axons. The neuronal localization of tau in cell soma when comparing human brain to neurons in culture might be explained by the culture system, the absence of neuronal connections and/or the environment surrounding neurons in brain. In order to see whether the cell body to axon redistribution of 4R tau could depend on its level of expression, hESC-derived neurons were transfected with the shortest 4R tau isoform at 21 DIV, before endogenous 4R was found in the axons. In transfected cells, the somatic distribution of 4R tau was abnormal for the age of the neurons, with 4R tau in both axons and cell bodies. Over-expression of 4R tau in hESC-derived neurons at 35 DIV, when they already have a high level of 4R tau that is present in both axons and cell bodies, did not show any clear effect on tau distribution. Axons of transfected cells showed increased fluorescence, suggesting increased presence of 4R tau although it is not possible to say if this involves endogenous 4R tau or transfected 4R tau or both. The expression of the F3 fragment alone increased endogenous tau phosphorylation as detected by AT8 staining. This increased phosphorylation was not due to an increase in tau levels as this was ruled out by staining with a total tau phosphorylation-independent antibody that showed equal amounts of tau in F3 transfected and non-transfected cells. The presence of the F3 molecule *per se* could not be responsible for the increased phosphorylation, because the F3 fragment does not contain the AT8 epitope. The co-expression of the F3 fragment and the shortest 4R tau isoform in hESC-derived neurons at 35 DIV promoted 4R tau redistribution and hyperphosphorylation, as indicated by AT8 and pSer262 staining. However, no staining with thioflavin S, FSB or AT100 was observed, suggesting that tau filaments were not present, nor was there any indication of toxicity, as shown by propidium iodide staining.

Together our results show that hESC-derived neurons represent a unique human model to study tau physiology and pathology. It should now be possible to apply this method of differentiation to neurons derived from induced pluripotent stem cells (iPSCs) from patients with tau pathologies and *Tau* gene mutations and to use these neurons for the screening of compounds to prevent tau dysfunction.
